# When to ask for an MRI of the scrotum

**DOI:** 10.1111/andr.13032

**Published:** 2021-06-11

**Authors:** Athina C. Tsili, Maria I. Argyropoulou, Miriam Dolciami, Giada Ercolani, Carlo Catalano, Lucia Manganaro

**Affiliations:** ^1^ Department of Clinical Radiology Faculty of Medicine School of Health Sciences University of Ioannina Ioannina Greece; ^2^ Department of Radiological, Oncological and Pathological Sciences Sapienza University of Rome Roma Italy

**Keywords:** magnetic resonance imaging, multiparametric magnetic resonance imaging, scrotum, testicular neoplasms, testis

## Abstract

**Background:**

Multiparametric MRI (mpMRI) of the scrotum has been established as a useful second‐line diagnostic tool for the investigation of scrotal diseases. Recently, recommendations on clinical indications for scrotal MRI were issued by the Scrotal and Penile Imaging Working Group of the European Society of Urogenital Radiology.

**Objective:**

To update current research on when to ask for an MRI of the scrotum.

**Methods:**

PubMed database was searched for original articles and reviews published during 2010–2021.

**Results:**

Eighty‐three articles fulfilled the search criteria. Scrotal MRI is mainly recommended after inconclusive US findings or inconsistent with the clinical examination and should be asked in the following cases: differentiation between intratesticular and paratesticular lesions (in rare cases of uncertain US findings), characterization of paratesticular and intratesticular lesions (when US findings are indeterminate), discrimination between germ cell and sex cord‐stromal testicular tumors, local staging of testicular malignancies (in patients planned for testis‐sparing surgery), differentiation between seminomas and non‐seminomatous tumors (when immediate chemotherapy is planned and orchiectomy is delayed), assessment of acute scrotum and scrotal trauma (rarely needed, in cases of non‐diagnostic US findings) and detection and localization of undescended testes (in cases of inconlusive US findings). Although preliminary data show promising results in the evaluation of male infertility, no established role for mpMRI still exists.

**Conclusion:**

Multiparametric MRI of the scrotum, by assessing morphologic and functional data represents a valuable problem‐solving tool, helping to improve our understanding on the nature of scrotal pathology and the process of spermatogenesis. The technique may improve patient care and reduce the number of unnecessary surgical procedures.

## 1 INTRODUCTION

Color Doppler ultrasonography (CDUS) represents the imaging modality of choice for the initial assessment of scrotal pathology.^1^, ^2^, ^3^, ^4^, ^5^, ^6^, ^7^, ^8^, ^9^ It is a safe, widely available, easily performed, inexpensive technique and does not use ionizing radiation. CDUS is highly sensitive and accurate in the assessment of scrotal diseases, often guiding proper treatment. Current guidelines, including the National Comprehensive Cancer Network and the European Association of Urology, advocate the use of US for the evaluation of a suspected testicular mass.^8^, ^10^

However, conventional US has limitations associated with operator‐dependence, relatively small field of view, and difficulties in tissue characterization. An accurate differentiation of the nature of scrotal lesions, especially lesions of small size is not always possible, based on sonographic features.^11^, ^12^, ^13^ Diagnoses such as a minor tear in the tunica albuginea in blunt scrotal trauma or chronic epididymoorchitis and partial or delayed torsion may sometimes be missed on sonography.^14^, ^15^, ^16^, ^17^, ^18^ The introduction of multiparametric US (including Color Doppler US) real‐time elastography and contrast‐enhanced US into clinical practice has improved the diagnostic performance of standard US in the investigation of scrotal diseases.^19^, ^20^, ^21^, ^22^, ^23^

Multiparametric MRI (mpMRI) of the scrotum has emerged as a valuable supplemental technique for the investigation of scrotal pathology.^7^, ^8^, ^9^, ^11^, ^12^, ^15^, ^24^, ^25^, ^26^, ^27^, ^28^, ^29^, ^30^, ^31^, ^32^, ^33^, ^34^, ^35^, ^36^, ^37^, ^38^, ^39^, ^40^, ^41^, ^42^, ^43^, ^44^, ^45^, ^46^ Scrotal MRI due to the wide field of view and multiplanar capabilities depicts in excellent anatomic detail both testes, epididymides, spermatic cords, and inguinal regions. The technique provides high soft‐tissue contrast, high sensitivity for contrast enhancement, and functional information, it is less dependent on operator compared to US and does not include ionizing radiation. Scrotal MRI allows differentiation between intratesticular and paratesticular lesions and accurate tissue characterization, by showing the presence of fat, hemorrhage, fibrosis, fluid content, and contrast‐enhancing tissue. MRI findings may narrow differential diagnosis, helping in planning more precise treatment strategies and reducing the need of unnecessary surgical explorations.^7^, ^8^, ^9^, ^11^, ^12^, ^15^, ^24^, ^25^, ^26^, ^27^, ^28^, ^29^, ^30^, ^31^, ^32^, ^33^, ^34^, ^35^, ^36^, ^37^, ^38^, ^39^, ^40^, ^41^, ^42^, ^43^, ^44^, ^45^, ^46^

Recently, the Scrotal and Penile Imaging Working Group (SPIWG) appointed by the board of the European Society of Urogenital Radiology (ESUR) has produced recommendations on clinical indications for scrotal MRI, based on literature published before 2016 and combined expertise of the group.^24^ MRI of the scrotum is primarily recommended for the characterization of paratesticular and intratesticular lesions in questionable cases, when US findings are indeterminate and for the identification and localization of undescended testes. The technique may provide valuable information in the pre‐operative planning, local staging, and histologic characterization of testicular germ cell neoplasms (TGCNs), in selected cases. It represents a supplemental, problem‐solving tool for the investigation of acute scrotum and scrotal trauma, following equivocal US findings.^24^ Scrotal MRI may prove reliable in differentiating between TGCNs and sex cord‐stromal tumors, specifically in characterizing Leydig cell tumors (LCTs), allowing the adoption of conservative surgery and active surveillance, in compliant patients, as treatment options.^24^, ^47^ The protocol of scrotal MRI should include axial T1‐weighted imaging (T1WI), axial and coronal T2‐weighted imaging (T2WI), axial diffusion‐weighted imaging (DWI), and coronal subtracted dynamic contrast‐enhanced (DCE) imaging.^24^

In this review, we summarize current research on when to ask for an MRI of the scrotum.

## 2 SEARCH CRITERIA AND STUDY SELECTION

Starting in January 2021, a structured search using PubMed database was performed and included all relevant original articles and reviews, published in and after 2010. The search used the following key word combinations: [(SCROTUM) OR (SCROTAL) OR (TESTICLE) OR (TESTICULAR) OR (PARATESTICULAR)] AND [(MRI) OR (Magnetic Resonance Imaging)] combined, depending on the specific domain of interest, with [(TUMOR) OR (TUMORAL) OR (CANCER) OR (MASS) OR (NEOPLASM)]; [(TRAUMA) OR (ACUTE) OR (TORSION) OR (EMERGENCY)]; [(CONGENITAL) OR (UNDESCENDED) OR (DESCENT) OR (NONPALPABLE)]; [(INFERTILITY) OR (FERTILITY) OR (STERILITY) OR (INFECUNDITY) OR (IMPOTENCE)]. Data extraction was independently performed by two reviewers (M.D. and G.E.), and any disagreement was discussed with a third reviewer (L.M.).

All papers published on human subjects were included. Citations and references of the retrieved studies were used as additional sources. Case reports, editorial comments, conference abstracts, and short communications were excluded.

## 3 RESULTS AND DISCUSSION

**FIGURE 1:**
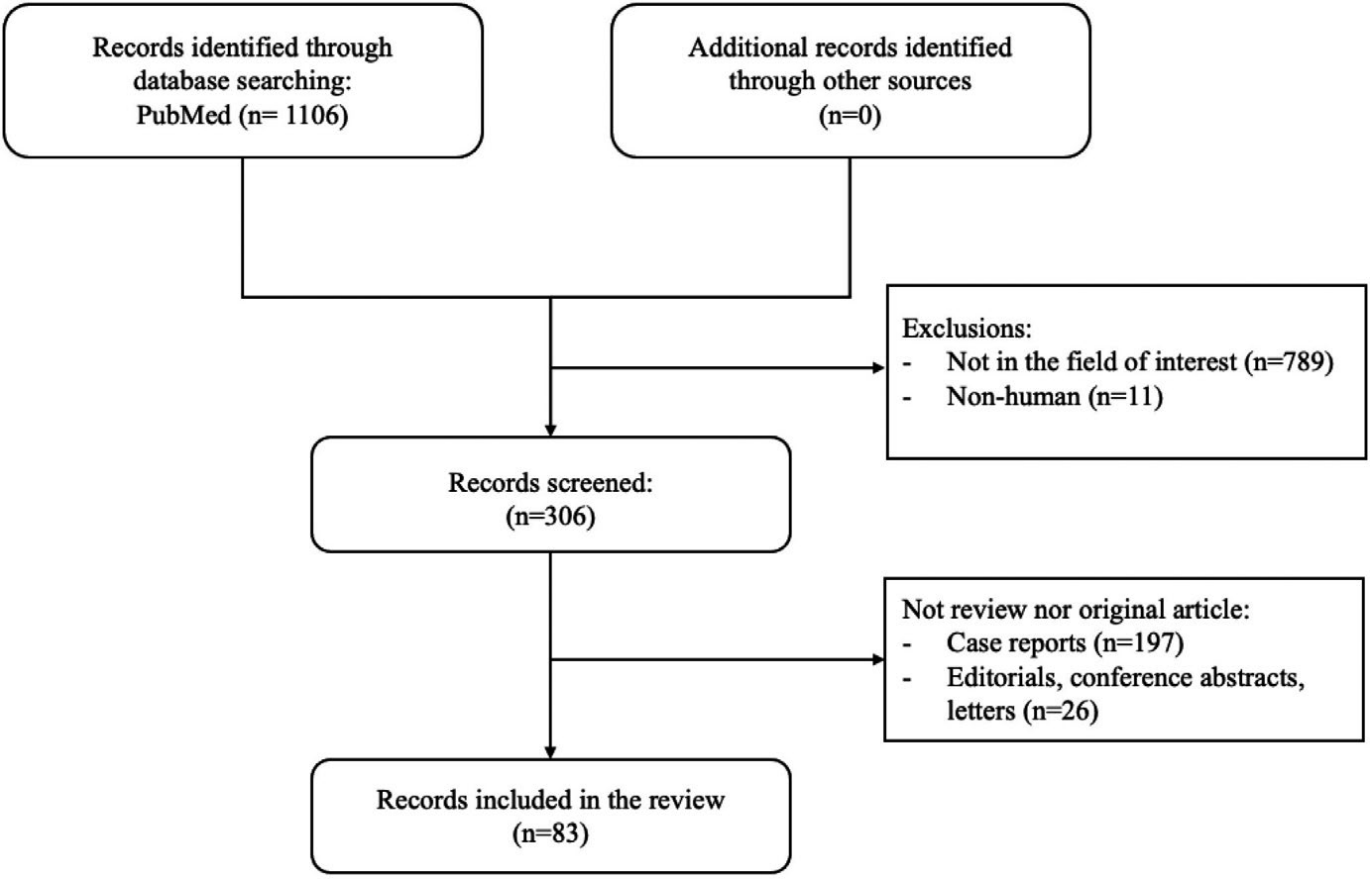
Flow chart showing study selection

The literature search found a total of 1106 articles (tumors, *n* = 584; acute scrotum, *n* = 193; undescended testes, *n* = 178; and infertility, *n* = 151). Ultimately, 83 articles were deemed relevant and used as the literature basis of this review (tumors, *n* = 50; acute scrotum, *n* = 6; undescended testes, *n* = 7; and infertility, *n* = 20).^6^, ^7^, ^8^, ^9^, ^13^, ^24^, ^25^, ^26^, ^27^, ^28^, ^29^, ^30^, ^31^, ^32^, ^33^, ^35^, ^43^, ^48^, ^49^, ^50^, ^51^, ^52^, ^53^, ^54^, ^55^, ^56^, ^57^, ^58^, ^59^, ^60^, ^61^, ^62^, ^63^, ^64^, ^65^, ^66^, ^67^, ^68^, ^69^, ^70^, ^71^, ^72^, ^73^, ^74^, ^75^, ^76^, ^77^, ^78^, ^79^, ^80^, ^81^, ^82^, ^83^, ^84^, ^85^, ^86^, ^87^, ^88^, ^89^, ^90^, ^91^, ^92^, ^93^, ^94^, ^95^, ^96^, ^97^, ^98^, ^99^, ^100^, ^101^, ^102^, ^103^, ^104^, ^105^, ^106^, ^107^, ^108^, ^109^, ^110^ The flow chart of the selection process is shown in Figure 1.

### 3.1 Main findings (indication for pathologies)

#### 3.1.1 Scrotal lesions

##### Lesion localization: intratesticular versus paratesticular

The determination of a scrotal lesion location is of outmost importance, since intratesticular solid mass lesions are malignant in more than 95% of cases and paratesticular mass lesions are more often benign. MRI is highly accurate in differentiating between intratesticular and paratesticular lesions, although rarely needed, since lesion localization is often a straightforward diagnosis for US.^24^

MRI is recommended in cases of questionable US findings, including patients with markedly enlarged scrotum.^25^ MRI also helps in the differentiation between a scrotal lesion originating from testicular tunica and peripheral seminiferous tubules, when this is difficult to define sonographically. The detection of a thin, hypointense, well‐defined halo, best depicted on T2WI, lying between the lesion and the adjacent testicular parenchyma, corresponding to the tunica albuginea, helps to suggest lesion origin.^5^, ^6^, ^7^, ^8^, ^24^, ^25^, ^29^, ^33^, ^42^, ^43^, ^44^, ^45^

MRI proved to be capable of identifying the correct lesion location with sensitivity, specificity, negative predictive value (NPV), and positive predictive value (PPV) of 100%, each, in a study including 84 patients with suspected scrotal masses.^35^

##### Paratesticular lesions

Paratesticular solid tumors are rare and more often benign, with an incidence of malignancy approximately of 3%. Accurate characterization of the nature of paratesticular lesions is of outmost importance to allow planning of a conservative treatment in benign lesions, such as surveillance, excision, or TSS. The US characteristics of paratesticular tumors are usually overlapping, and therefore, scrotal MRI is strongly recommended as a valuable supplemental technique, to define lesion location and extent and to suggest a possible histologic diagnosis.^6^, ^8^, ^29^, ^30^, ^33^, ^36^, ^42^, ^43^, ^44^, ^45^

Although the sonographic diagnosis of an epididymal cyst or a tunica albuginea cyst is often straightforward, concerns may arise in the presence of complex cysts. In these cases, absence of enhancement on MRI confirms an avascular mass and the diagnosis of benignity. MRI with multiplanar imaging also helps in suggesting lesion location.^30^, ^36^

**FIGURE 2:**
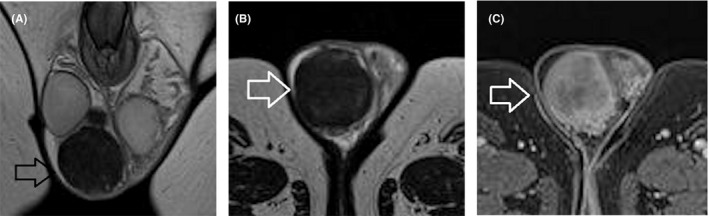
Adenomatoid tumor of the epididymis. T2WI in (A), coronal and (B), transverse planes show a large, well‐defined right paratesticular mass (arrow), of low signal. (C) Transverse post‐contrast T1WI depicts lesion (arrow) enhancing heterogeneously

When differentiation between an adenomatoid tumor and a peripheral intratesticular mass is ambiguous on US, scrotal MRI is highly recommended to suggest paratesticular origin. Surgery in paratesticular adenomatoid tumors includes intraoperative frozen section biopsy and local resection, whereas in intratesticular adenomatoid tumors, TSS is planned.^6^, ^8^, ^29^, ^33^, ^36^, ^43^, ^44^, ^111^ On MRI, adenomatoid tumor usually appears hypointense on T2WI, with slow or decreased contrast enhancement, relative to the normal testis (Figure 2). ^30^, ^36^, ^111^

MRI is helpful to suggest the paratesticular location and the benign nature of fibrous pseudotumor. Sonographic findings of this benign lesion are usually non‐specific and may guide to an unnecessary radical orchiectomy. Lesion hypointensity on both T1WI and T2WI, with slow but persistent enhancement, characteristics for the presence of fibrous tissue allows for an accurate diagnosis. Local excision and frozen section confirmation may be recommended, following scrotal MRI.^6^, ^8^, ^29^, ^30^, ^36^, ^44^, ^112^, ^113^, ^114^

US is not always suggestive of the diagnosis of paratesticular lipoma, as a hyperchoic appearance cannot be easily differentiated from other paratesticular lesions, including lymphadenopathy, inguinal hernia, or liposarcoma. Moreover, lipomas may appear with variable echogenicity on sonography. MRI provides a specific diagnosis, as the lesion shows fat signal characteristics, with lack of enhancement. The presence of chemical shift artifact within or at the margins of the lesion can be used to confirm lipomatous content. MRI also helps in confirming the paratesticular location of large‐sized lipomas.^6^, ^8^, ^29^, ^30^, ^36^, ^42^, ^43^, ^44^

MRI is extremely helpful in suggesting the pre‐operative diagnosis of a paratesticular sarcoma and in evaluating the local extent of the disease. Paratesticular sarcomas are usually ill‐defined, heterogeneous solid masses, strongly and inhomogeneously enhancing, with areas of restricted diffusion.^6^, ^8^, ^42^, ^43^, ^44^ The presence of macroscopic fat easily appreciated on MRI is suggestive of a well‐differentiated paratesticular liposarcoma.

MRI characteristics may suggest the histologic nature of other extremely rare paratesticular tumors.^30^, ^43^ The diagnosis of a scrotal hemangioma is possible when a dominant feeding or draining vessel accompanies a markedly hyperintense scrotal lesion on T2WI, especially in the presence of foci of signal void, corresponding to phleboliths. MRI is valuable in assessing both the multicystic nature and the local extent of a lymphangioma, before planning surgical intervention. Perineal aggressive angiomyxoma should be included in the differential diagnosis, in the presence of hyperintense T2 signal due to myxoid stroma and a whorled pattern of T2 signal.^43^ MRI helps in the identification of the solid component of a mesothelioma of the tunica vaginalis, which can manifest as multiple, small nodules on the surface of the tunica vaginalis or as diffuse thickening, with vegetations. These lesions often show low T2 signal and hypervascularity.^61^

MRI performs better than US in the identification of the contents of an inguinal hernia and also in the detection of symptomatic hernias, which are impalpable. Fat‐containing hernias appear as elongated masses arising from the inguinal canal, with signal intensity similar to that of subcutaneous fat.^30^, ^115^

MRI is recommended to confirm the presence of polyorchidism, in cases of indeterminate US findings, and to provide additional information in complicated cases, including cryptorchidism and testicular neoplasms. MRI shows a rounded or oval lesion, with signal characteristics and enhancement patterns often similar to normal testis. The detection of the tunica albuginea, as a hypointense T2 rim surrounding the supernumerary testis, confirms the diagnosis. Occasionally, the mediastinum testis, supernumerary epididymis, and bridging vessels between the normal testis and the extra‐testicle may be seen.^6^, ^8^, ^29^, ^30^, ^44^, ^116^, ^117^ Table 1 shows common paratesticular lesions for which mpMRI is useful.

**TABLE 1:** Common paratesticular lesions: when to ask for an MRI of the scrotum

Paratesticular lesions	When to ask for scrotal MRI?	MRI advantages	MRI findings
Epididymal or tunica albuginea cyst	Rarely needed, in cases of complex cysts	Specific diagnosis	Well‐defined, homogeneous, watery signal, absence of solid elements and enhancement
Adenomatoid tumor	Differentiation from an intratesticular mass with peripheral location	Paratesticular location	Well‐defined, low T2 signal, slow or mild enhancement
Fibrous pseudotumor	Often indeterminate US findings	Highly suggestive/lesion localization	Low T2 signal, low, persistent enhancement
Lipoma	Indeterminate US findings	Specific diagnosis/lesion localization	Signal characteristics similar to fat, lack of enhancement
Sarcoma	Compliments US	Suggestive of diagnosis/local extent	Ill‐defined, heterogeneous, contrast‐enhancing components, restricted diffusion; presence of fat denotes liposarcoma
Hemangioma	Indeterminate US findings	Suggestive of diagnosis	Markedly hyperintense on T2WI, foci of signal void (phleboliths), dominant feeding or draining vessel
Lymphangioma	Indeterminate US findings	Suggestive of diagnosis/disease extent	Multicystic
Perineal aggressive angiomyxoma	Indeterminate US findings	Suggestive of diagnosis	Hyperintense on T2WI (myxoid stroma), whorled pattern
Mesothelioma of tunica vaginalis	Indeterminate US findings	Suggestive of diagnosis	Diffuse thickening of testicular tunica, low T2 signal, contrast‐enhancing
Inguinal hernia	Indeterminate US findings	Identification of hernia contents/detection of impalpable symptomatic hernias	Elongated mass, signal characteristics similar to fat
Polyorchidism	Indeterminate US findings	Specific diagnosis/complications	Rounded or oval, signal characteristics and enhancement patterns often similar to normal testis, surrounded by a low T2 rim (tunica albuginea). Rarely seen: mediastinum testis, bridging vessels, supernumerary epididymis

##### Intratesticular lesions

###### Lesion characterization: benign versus malignant

The great majority of solid intratesticular lesions are malignancies, with TGCNs representing approximately 95% of testicular carcinomas.^118^ Although rare, benign intratesticular entities include a wide variety of both non‐neoplastic pathologies, such as testicular cyst, tubular ectasia of rete testis (TERT), fibrosis, hematoma, segmental testicular infarction (STI), intratesticular lipoma Leydig's cell hyperplasia, and adrenal rest tumors.^11^ Accurate characterization of the nature of benign intratesticular lesions greatly improves patient management and reduces the need of radical surgery. Alternative treatments, such as follow‐up, biopsy, lesion enucleation, and testis‐sparing surgery (TSS) may be proposed.^11^ Scrotal mpMRI is highly recommended for the characterization of intratesticular lesions, in cases of equivocal US findings.^24^ MRI is highly sensitive in the characterization of benign intratesticular lesions, by depicting the presence of fluid, fat, fibrous tissue, blood products, myxoid, and granulation tissue.^6^, ^7^, ^8^, ^9^, ^11^, ^12^, ^14^, ^24^, ^25^, ^26^, ^27^, ^28^, ^29^, ^30^, ^33^, ^34^, ^35^, ^36^, ^37^, ^42^, ^48^, ^50^, ^51^, ^52^, ^53^, ^56^ The overall accuracy of MRI in the characterization of benign testicular diseases has been reported 87.5%, and the NPV 100%.^48^ The absence of contrast enhancement represents a highly sensitive sign in characterizing the benign nature of intratesticular lesions.^6^, ^48^

The sonographic findings of testicular cysts and TERT are usually typical, and therefore, MRI is rarely needed. However, it is recommended when differential diagnosis from cystic neoplasms, including cystic teratomas and papillary adenocarcinomas of the rete testis is difficult.^7^, ^11^, ^29^, ^30^, ^36^, ^119^ Typical MRI criteria, including the presence of tubular cystic structures, of watery signal, involving the mediastinum testis, with absence of solid elements and contrast enhancement, are used for the diagnosis of TERT.^7^, ^11^, ^29^, ^30^

The sonographic diagnosis of testicular fibrosis is usually difficult. MRI is recommended as a confirmatory study. The low T1 and T2 signal of fibrous tissue and the slow, progressive contrast enhancement are highly suggestive findings.^25^

Hypoechoic intratesticular hematomas, especially in cases of uncertain history of trauma may be difficult to differentiate from malignant tumors. Even in the absence of lesion vascularity on CDUS, the diagnosis of an underlying malignancy cannot be excluded. MRI confirms the presence of a hematoma, when a prompt diagnosis is needed, by showing lesion hyperintensity on T1WI in the subacute phase, with lack of enhancement. A T2 hypointense rim may be detected on chronic hematomas, due to hemosiderin deposition.^29^, ^30^, ^36^

MRI is highly accurate in establishing the diagnosis of STI, mainly recommended in cases with indeterminate sonographic findings, such as those of a rounded, ill‐defined hypoechoic intratesticular lesion, with uncertain vascularity. MRI findings include an avascular lesion, mainly of low T2 signal, and a markedly enhancing rim surrounding the mass. Occasionally, intralesional hyperintense T1 foci may coexist, due to hemorrhagic elements.^8^, ^29^, ^30^, ^52^, ^120^ Short‐interval sonographic follow‐up is recommended to allow a confident diagnosis.

Although intratesticular lipomas are typically hyperechoic on US, their histologic complexity may result in variable echogenicity. In these cases, MRI findings are diagnostic, as lipoma follows the signal intensity characteristics of fat on all sequences, with lack of contrast enhancement. Scrotal MRI by depicting multiple, small hyperintense intratesticular foci on T1WI, with drop of signal of fat‐saturated T1WI confirms the diagnosis of testicular lipomatosis, a rare entity, seen in patients with Cowden disease.^6^, ^29^

Scrotal MRI is recommended for the diagnosis of Leydig's cell hyperplasia. The detection of multiple, bilateral small intratesticular lesions, of few millimeters in diameter, hypointense on T2WI, with mild contrast enhancement combined with the appropriate clinical history and laboratory data, is strongly suggestive of the diagnosis of this benign entity. Compared to sonography, MRI may demonstrate more lesions and confirm bilaterality.^11^, ^29^, ^34^, ^121^

MRI is helpful for the diagnosis of testicular adrenal rest tumors, strongly recommended in candidates for TSS. These lesions are often bilateral, involve the mediastinum testis, of low T2 signal, variably enhancing after gadolinium administration. MRI surpasses US, by assessing the extent of the disease. Lesion size and margins are clearly delineated and differentiation between different small‐sized contiguous foci and one, large lobular lesion is possible.^11^, ^29^, ^30^, ^122^, ^123^ Table 2 shows common benign intratesticular lesions for which mpMRI is useful.

**TABLE 2:** Common benign intratesticular lesions: when to ask for an MRI of the scrotum (TSS, testis‐sparing surgery)

Benign intratesticular lesions	When to ask for scrotal MRI?	MRI advantages	MRI findings
Non‐neoplastic			
Testicular cyst	Rarely needed; when differentiation from cystic tumors is difficult at US	Specific diagnosis	Well‐defined, homogeneous mass, watery signal, absence of solid components and enhancement
Testicular ectasia of rete testis	Rarely needed; when differentiation from cystic tumors is difficult at US	Specific diagnosis	Tubular cystic structures in mediastinum testis, watery signal, lack of enhancement
Fibrosis	Often indeterminate US findings	Highly suggestive	Low T1, T2 signal, slow, progressive enhancement
Hematoma	Indeterminate US findings	Suggestive of diagnosis	T1 hyperintensity (subacute phase), hypointense T2 rim (chronic phase), absence of enhancement
Segmental testicular infarction	Indeterminate US findings	Suggestive of diagnosis	Low T2 signal, contrast‐enhancing rim. May have hyperintense T1 areas, triangular shape, pointing toward mediastinum testis
Benign neoplastic			
Lipoma	Indeterminate US findings	Specific diagnosis	Signal characteristics similar to fat, no enhancement
Leydig's cell hyperplasia	Compliments US	Depicts more foci and confirms bilaterality	Multiple, bilateral foci, of few mm, low T2 signal, mild enhancement
Adrenal rest tumors	Candidates for TSS	Disease extent	Multiple, bilateral masses, low T2 signal, variable enhancement, involving mediastinum testis

**FIGURE 3:**
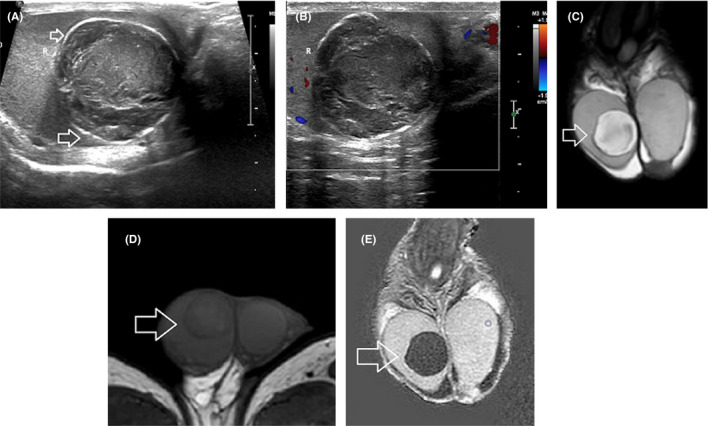
Epidermoid cyst. (A) Gray‐scale image depicts a solid, heterogeneous, intratesticular mass, of laminated appearance. The lesion is surrounded by a thin echogenic rim (small arrows). (B) Color Doppler image shows absence of internal vascularity. (C) Coronal T2WI demonstrates right intratesticular mass lesion (arrow), with heterogeneous signal, mainly hyperintense, encircled by a hypointense halo. (D) Axial T1WI demonstrates lesion (arrow) internal heterogeneity. (E) Coronal subtracted DCE image depicts absence of lesion vascularity (arrow), a finding confirming the diagnosis of benignity

Based on the recent WHO classification, testicular epidermoid cyst (EC) is considered a subtype of teratoma of prepubertal type, included in the group of TGCNs unrelated to germ cell neoplasia in situ.^118^ Lesion enucleation may be suggested for ECs <3 cm and typical imaging findings, in patients with negative tumor markers, although intraoperative frozen section is needed to confirm the diagnosis.^7^ MRI may provide confirmatory findings and is mainly recommended as a second‐line tool in cases of equivocal US findings. MRI findings specific for the diagnosis of an EC include the presence of a rounded or oval encapsulated intratesticular lesion, surrounded by a hypointense T2 halo, not enhancing after gadolinium administration (Figure 3), with the characteristic “onion skin sign” appearance on T2WI (alternating concentric rings of high and low T2 signal) or a target appearance (due to a hyperintense central area on T1WI).^7^, ^11^, ^29^, ^36^, ^37^, ^70^, ^71^, ^124^, ^125^

The minimum requirements of scrotal MRI protocol include the addition of DWI and DCE‐MRI.^24^

**FIGURE 4:**
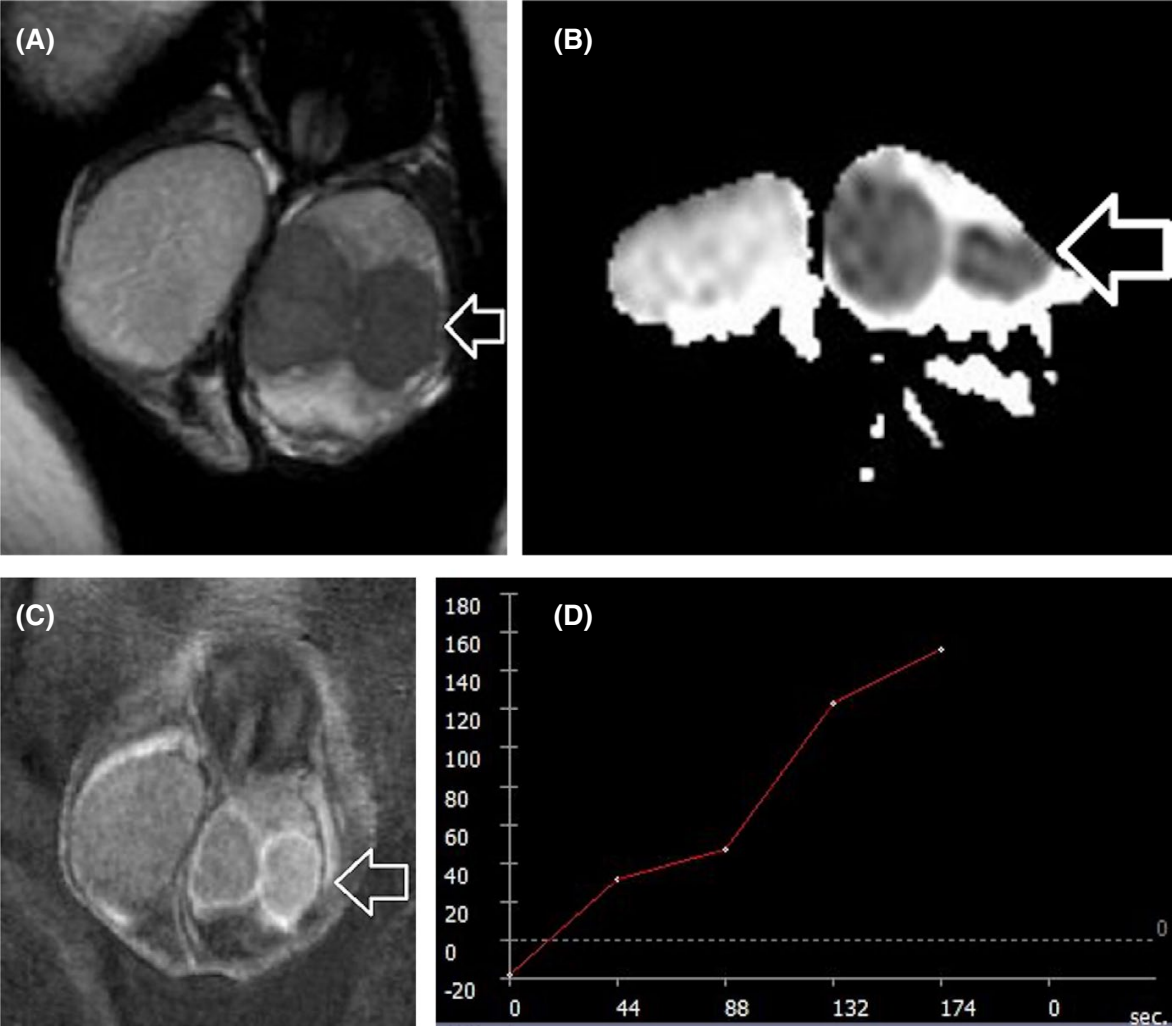
Typical testicular seminoma. (A) Coronal T2WI shows a multilobular left intratesticular tumor (arrow), mainly homogeneous, of low signal. (B) Transverse ADC map. The tumor (arrow) appears hypointense, due to diffusion restriction. The mean ADC of seminoma is 0.51 × 10^−3^ mm^2^/s. (C) Coronal subtracted DCE image depicts tumoral septa enhancing more than the remaining neoplasm (arrow). (D) TSI curve of the tumor. Testicular seminoma enhances early and avidly (curve type III)

DWI by assessing the microscopic diffusion movements of water molecules in testicular tissue, greatly improves the diagnostic efficiency of MRI in the detection and characterization of intratesticular lesions.^50^, ^51^, ^55^, ^56^, ^57^, ^63^, ^72^ Testicular malignancies often present with restricted diffusion and a lower apparent diffusion coefficient (ADC) value, when compared to normal testis and benign intratesticular lesions (Figure 4). A cut‐off ADC of 0.99 × 10^−3^ mm^2^/s has been reported reliable for the characterization of intratesticular lesions, with a sensitivity of 93.3%, specificity of 90%, PPV of 87.5%, and NPV of 94.7%.^56^

By manually segmenting the whole testicular lesion on the ADC map, volumetric ADC histogram analysis was used to characterize 61 pathologically confirmed intratesticular lesions. An increase in energy, total energy, and range of ADC and a decrease in minimum ADC and 10th percentile ADC was observed in testicular neoplasms, compared to benign intratesticular lesions. The minimum ADC had the highest diagnostic performance in characterizing testicular lesions, with an area under the curve (AUC) of 0.822, sensitivity of 81.40%, and specificity of 77.78%.^75^

Moreover, intratesticular lesions have increased anisotropy, when compared to normal testicular parenchyma. This was proved in a recent preliminary study, using diffusion tensor imaging (DTI) to characterize testicular mass lesions and reporting high fractional anisotropy (FA) values in both TGCNs and benign lesions.^66^

The patterns of contrast enhancement on dynamic imaging represent an adjunct parameter in differentiation of intratesticular lesions.^14^, ^53^, ^57^ Significant differences in time‐signal intensity (TSI) curves between healthy testicular tissue, benign, and malignant lesions have been presented.^53^, ^57^ Normal testes usually enhance homogeneously, with a gradual increase in signal intensity at DCE‐MRI (type I curve). Benign intratesticular lesions show either lack of contrast enhancement (type 0 curve) or a homogeneous/heterogeneous early, avid enhancement, followed by a plateau or a gradual further enhancement (type 2 curve).^53^, ^57^ Type 3 curve is often seen in TGCNs, detected as an early, strong enhancement, followed by a gradual de‐enhancement (Figure 4).^53^, ^57^

Although published data are limited, preliminary observations on other MRI techniques, including magnetization transfer imaging (MTI), proton MR spectroscopy (1H‐MRS), and texture analysis report useful results in the characterization of the nature of intratesticular lesions.^58^, ^62^, ^78^ TGCNs have high magnetization transfer ratio (MTR) when compared to normal testis and benign testicular lesions, a finding related to an increase in macromolecular content.^62^ Proton MRS had a sensitivity and specificity of 80% in differentiating normal testes from a variety of testicular diseases. The same study reported a decrease in choline levels, seen in three testicular tumors (Figure 6).^58^

Recently, texture analysis was used to characterize testicular lesions. Specifically, histogram analysis and intra‐perinodular textural transition (Ipris) were applied, after manually segmenting testicular lesions on T2WI. Twelve significantly different features were found between benign and malignant tumors, of which the most robust were Energy, Total Energy, and Ipris_shell1_id_std with AUC of 0.807, 0.808, and 0.708, respectively.^78^

###### Germ cell versus sex cord‐stromal testicular tumors

The increased use of scrotal US has resulted in a high incidence of small, impalpable solid testicular mass lesions, detected as incidental findings. Histologic diagnosis is benign in approximately 80% of these cases, and LCTs represent the commonest pathology.^47^, ^126^ TSS is highly recommended in these patients. When a sex cord‐stromal tumor is suggested by frozen section examination, radical orchiectomy may be avoided.^10^, ^47^, ^127^

**FIGURE 5:**
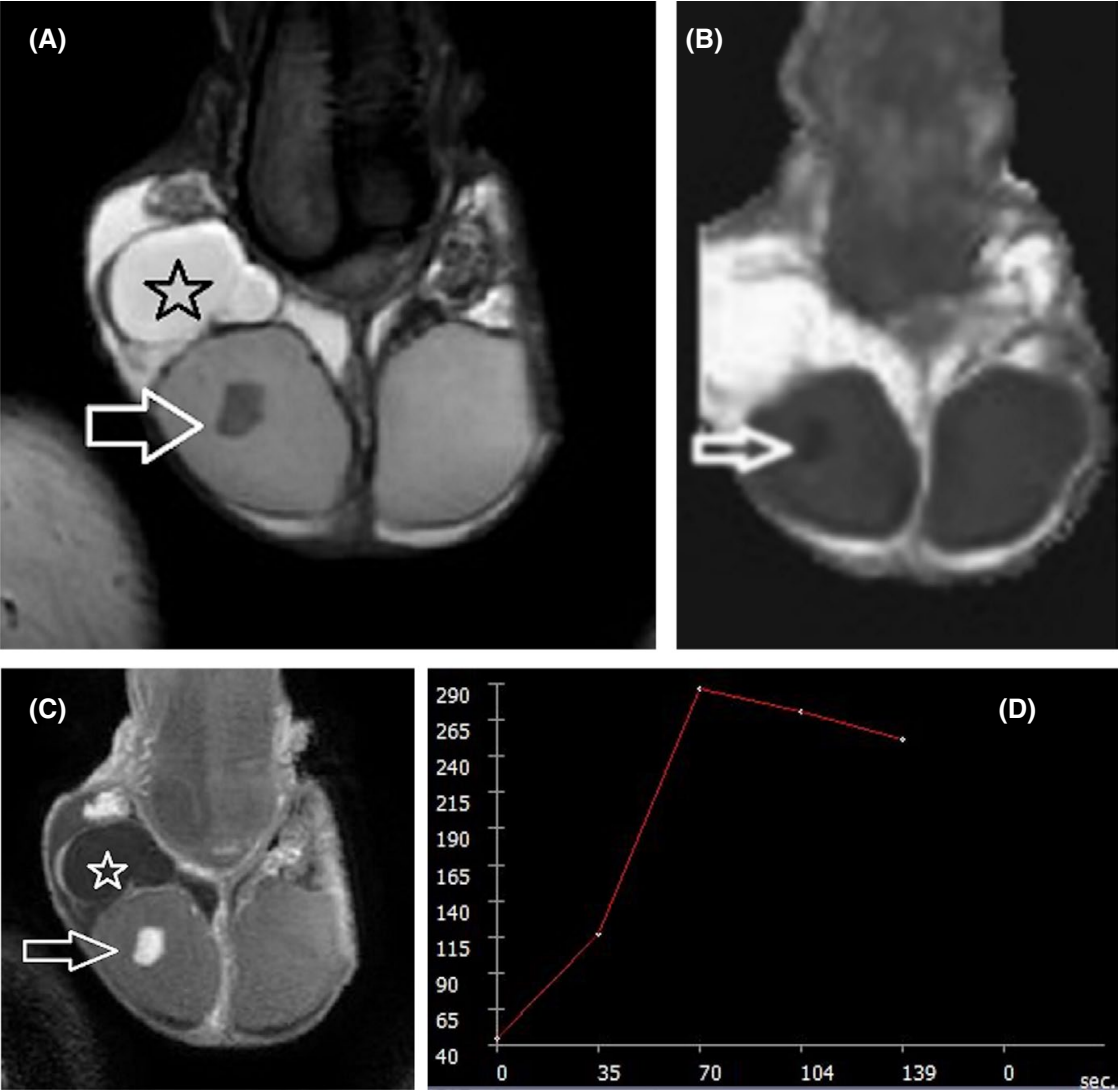
Leydig cell tumor. (A) Axial T2WI demonstrates small right intratesticular mass (arrow), of low signal. The maximal lesion diameter is 11 mm. (B) Coronal ADC map depicts lesion diffusion restriction (arrow). The mean ADC of the lesion is 0.77 × 10^−3^ mm^2^/s, lower than that of the normal contralateral testis (1.06 × 10^−3^ mm^2^/s). (C), Coronal subtracted DCE image and D, TSI curve. The lesion demonstrates strong, early, homogeneous contrast enhancement, with rapid de‐enhancement (curve type III, D). An ipsilateral spermatocele is also seen in the right paratesticular space as a well‐defined multicystic lesion, of watery signal (asterisk, A, C)

Although no established imaging criteria exist, mpMRI may help in the characterization of LCTs and in the differentiation from TGCNs, and especially testicular seminomas.^6^, ^7^, ^13^, ^24^, ^47^, ^59^, ^68^, ^77^ MRI features suggesting the diagnosis of LCTs include a well‐defined, intratesticular mass, markedly hypointense on T2WI, homogeneously enhancing after gadolinium administration, with early, strong enhancement, followed by slow de‐enhancement (Figure 5).^13^ MRI characteristics of testicular seminomas include an ill‐defined tumor, slightly hyperintense, and hypointense on T1WI and T2WI, respectively, with gradual contrast enhancement and absence of de‐enhancement (Figure 4).^13^

Semiquantitative and quantitative DCE‐MRI parameters have been reported useful in the characterization of small, impalpable solid testicular tumors.^68^ LCTs have lower percentage of peak enhancement, wash‐in‐rate, volume transfer constant, and rate constant, and shorter time to peak when compared to seminomas.^68^ Moreover, a recent retrospective study reported that benign testicular stromal tumors have smaller size, more hypointense T2 signal, higher ADC, and more homogeneous enhancement, when compared to malignant stromal and non‐stromal testicular tumors.^77^

##### Local staging and histologic characterization of TGCNs

Radical inguinal orchiectomy, with removal of the entire testis containing tumor along with the spermatic cord to the level of the internal inguinal ring, is the treatment of choice for testicular malignancies and should be performed within a week of initial diagnosis.^7^, ^10^ TSS with frozen section examination may be attempted in patients with a solitary testis.^10^ Accurate estimation of the local extent of TGCNs is important in candidates for TSS.

MRI provides valuable information regarding local staging of testicular malignancies, such as tumor dimensions, possible invasion of the rete testis, the testicular tunicae, the paratesticular structures, and/or the spermatic cord.^9^, ^19^, ^30^, ^34^, ^35^, ^36^, ^37^, ^48^ Tumor pseudocapsule detected as a hypointense rim surrounding malignancy on T2WI has been described as a feature facilitating TSS.^48^ Based on the results of a retrospective study, including 28 TGCNs, MRI correctly assessed the local extent of the disease in 92.8% of cases.^48^

**FIGURE 6:**
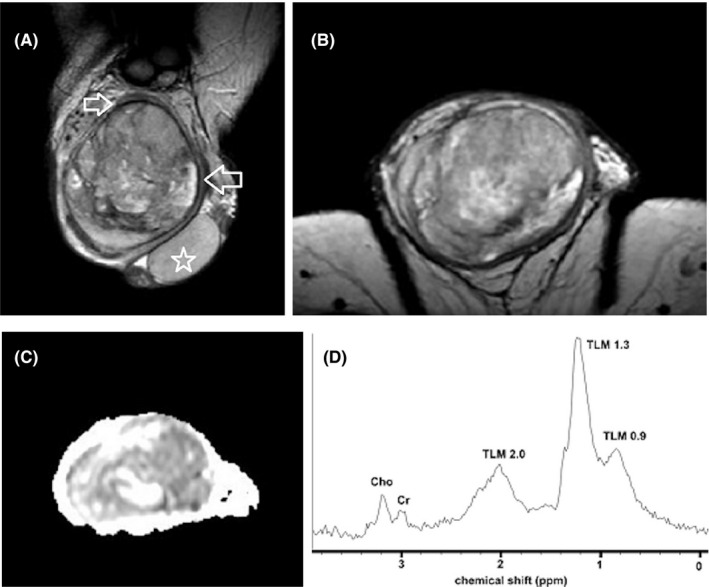
Embryonal carcinoma of the right testis. T2WI in (A), coronal and (B), transverse planes depict a large heterogeneous right testicular tumor. The mass is surrounded by a hypointense rim (small arrows), proved to correspond to tumor pseudocapsule on pathology. Left normal testis (asterisk). (C) Axial ADC map. The mean ADC of the tumor is 1.08 × 10^−3^ mm^2^/s. D, Proton MR spectrum of testicular neoplasm shows significant decrease in choline peaks (Cho: choline; Cr: creatine; TLM 2.0 ppm: total lipids and macromolecules resonating at 2.0 ppm; TLM 1.3: total lipids and macromolecules resonating at 1.3 ppm; and TLM 0.9 ppm: total lipids and macromolecules resonating at 0.9 ppm)

Multiparametric MRI features closely correlate with the histologic characteristics of TGCNs.^30^, ^36^, ^57^, ^63^, ^128^, ^129^ Typically, seminomas are detected as multilobular tumors, mainly homogenous and hypointense on T2WI. Fibrovascular septa are often seen within seminomas, as hypointense T2 bands, enhancing more than the remaining tumor, after gadolinium administration (Figure 4). Non‐seminomatous GCNs are usually heterogeneous, with inhomogeneous enhancement.^128^, ^129^ A hypointense rim, corresponding to fibrous capsule on pathology is seen more common on non‐seminomas (Figure 6).^129^ ADC is also efficient in characterizing TGCNs. Seminomas usually have lower ADC, when compared to non‐seminomas (Figures 4 and 5).^57^ A cut‐off ADC of 0.68 × 10^−3 ^mm^2^/s is reliable in differentiating seminomas from non‐seminomatous tumors.^63^ Table 3 shows common testicular neoplasms for which mpMRI is useful.

**TABLE 3:** Common testicular neoplasms: when to ask for an MRI of the scrotum (TSS, testis‐sparing surgery)

	When to ask for scrotal MRI?	MRI advantages	MRI findings
Testicular germ cell neoplasms			
Lesion characterization	Indeterminate US findings	Highly suggestive	Low or heterogeneous T2 signal, restricted diffusion, inhomogeneous enhancement, type III curve
Local staging	Candidates for TSS	Highly suggestive	Tumor dimensions, tumor pseudocapsule, invasion of rete testis, testicular tunicae, paratesticular structures, and/or spermatic cord
Differentiation between seminomas and non‐seminomas	Rarely needed; when chemotherapy is the recommended primary treatment (in cases of extensive metastases)	Highly suggestive	*Seminoma*: Lobular, homogeneous, and hypointense on T2WI, septa of low T2 signal, enhancing more than the remaining tumor, lower ADC, compared to non‐seminoma
			*Non*‐*seminoma*: heterogeneous on T1WI and T2WI, inhomogeneous enhancement, often surrounded by a hypointense halo
Epidermoid cyst	Indeterminate US findings	Highly suggestive	Round or oval, well‐defined, surrounded by a hypointense halo on T2WI, onion skin or target appearance, lack of enhancement
Leydig cell tumor	Indeterminate US findings	may help in diagnosis	Small size, well‐defined, markedly hypointense on T2WI, strong, early, homogeneous enhancement

Recently, whole‐lesion ADC histogram analysis and T2WI‐based radiomics provided promising results in the characterization of the histologic type of TGCNs.^69^, ^79^ A significant reduction in the median 10th, 25th, 50th, 75th, and 90th percentiles and the mean, minimum, and maximum ADC, and a significant increase in the median kurtosis and skewness of ADC was found in seminomas compared with non‐seminomas, in a retrospective study of 24 TGCNs.^69^ In particular, the 10th percentile ADC yielded the highest AUC, with a sensitivity and specificity of 100% and 92.86%, respectively.^69^ T2WI‐based radiomics also proved helpful to discriminate between seminomas and non‐seminomas.^79^

Characterization of the histologic type of TGCNs is rarely needed, in patients diagnosed with disseminated disease and/or life‐threatening metastases, in whom immediate chemotherapy is given. In these cases, MRI is strongly recommended.^24^, ^30^, ^36^

#### 3.1.2 Acute scrotal diseases

CDUS remains the first imaging technique for the investigation of acute scrotum, and specifically for the differentiation between testicular torsion and acute epididymoorchitis.^1^, ^2^, ^4^, ^130^, ^131^ Although rarely needed, scrotal MRI may be used as a helpful supplemental diagnostic tool. The technique is mainly recommended in cases of equivocal US findings.^12^, ^14^, ^15^, ^18^, ^24^, ^25^, ^28^, ^38^

##### Acute scrotum

Testicular torsion represents a real surgical emergency. The detection of testicular blood flow on CDUS may be limited in some cases, depending on operator expertise and US machine sensitivity, and this is problematic, especially in prepubertal testes.^14^, ^15^ Subtracted DCE‐MRI is highly sensitive and specific for the diagnosis of testicular torsion, by detecting decrease or lack of testicular perfusion.^14^, ^132^, ^133^ Moreover, DWI may allow the diagnosis of testicular torsion, without the use of intravenous contrast media. A lower ADC is observed in the twisted testis compared to the contralateral normal testis.^17^

Recently, MRI has been reported helpful in the diagnosis of bell clapper deformity (BCD), an important risk factor for testicular torsion. The detection of BCD may also help in diagnosing intermittent testicular torsion. The “split sign” has been described as useful in diagnosing BCD, detected as a hyperintense T2 area placed between the posterior aspect of the epididymis and the scrotal wall.^83^

Multiparametric MRI may complement the sonographic evaluation in patients with acute scrotal pain due to testicular torsion, as a confirmatory examination, helping to determine when and how to perform surgical intervention.^14^, ^24^ The detection of diminished or absent testicular perfusion at MRI, associated with pathologic T2 signal suggests the diagnosis of testicular torsion with hemorrhagic necrosis, and therefore, orchiectomy should be followed. Surgical untwisting and orchiopexy should be performed in testes with hypoperfusion or lack of perfusion and normal T2 signal.^14^

Scrotal MRI is also helpful in the diagnosis of incomplete testicular torsion or delayed torsion. Both entities usually have misleading characteristics, on clinical and US evaluation.^12^, ^16^, ^37^

MRI may be used as a second‐line imaging examination in the assessment of complicated scrotal infections and inflammations. Scrotal MRI provides a reliable map, regarding the extent of the disease, especially when there is concern for Fournier's gangrene, helping in planning percutaneous drainage or surgical debridement.^8^, ^18^, ^28^, ^43^ The technique also may be used to suggest the diagnosis of a scrotal abscess, occasionally difficult to differentiate from other testicular pathologies, including testicular torsion, hematoma, or tumor.^28^, ^43^ At MRI, abscess displays a hyperintense T2 signal, peripheral enhancement, and markedly restricted diffusion. MRI also enables excellent depiction and mapping of scrotal skin or perineum sinus tracts or fistulous tracts.^28^, ^43^, ^134^

MRI limitations in the assessment of acute scrotum should be acknowledged, including limited availability of an urgent MRI study and possible need for anesthesia in young patients.

##### Scrotal trauma

MRI is rarely needed in cases of scrotal trauma, as sonography is often diagnostic.^1^, ^2^, ^4^, ^5^, ^24^, ^135^

However, in cases equivocal for the diagnosis of testicular rupture at US, MRI represents a valuable adjunct tool. Multiplanar T2WI greatly assesses the integrity of the tunica albuginea, in patients with blunt scrotal trauma.^18^, ^24^, ^81^, ^135^

MRI may be proposed for the differentiation between a post‐traumatic hematoma and a TGCN, considering that testicular malignancies represent an incidental US finding in 15% of men presenting with scrotal trauma. Lack of enhancement confirms benignity in these cases, allowing the distinction from hypovascular tumors.^18^, ^81^, ^135^ Moreover, due to the large field of view, MRI may provide additional valuable information in extensive traumatic injuries and also in cases of a dislocated testis.^8^, ^18^, ^39^, ^81^, ^135^

#### 3.1.3 Undescended testes

US and MRI have similar accuracy in the identification of undescended testes, although the sensitivity of MRI has been reported superior to that of sonography.^7^, ^136^ Based on the recent ESUR guidelines, MRI is highly recommended for the identification and localization of undescended testes, especially intraabdominal testes, in cases of uncertain sonographic results.^7^, ^24^, ^35^, ^36^, ^42^ The detection of gubernaculum and/or the spermatic cord is ancillary findings, helping to confirm the diagnosis. MRI is also useful in discriminating between an undescended testis and testicular agenesis.^7^, ^35^, ^36^, ^42^

DWI and T2WI with fat saturation increase the diagnostic performance of MRI in the detection and localization of impalpable, undescended testes.^24^ Information on testis viability may be provided at DWI, with testes of low DWI signal considered nonviable.^84^, ^85^, ^89^

Interestingly, a fetal MRI study investigating the time course of testicular descent in utero according to gestational age, highlighted the importance of assessing male sexual development and early diagnosis of congenital anomalies, such as cryptorchidism.^90^

#### 3.1.4 Infertility

Recently, scrotal MRI has focused on the evaluation of deranged spermatogenesis. Although reported data are still preliminary and heterogeneous, mainly based on small sample size, various MRI parameters, including ADC, FA, MTR, and testicular metabolites have been described as possible non‐invasive fingerprints of male infertility.^91^, ^92^, ^93^, ^94^, ^95^, ^96^, ^97^, ^98^, ^99^, ^100^, ^101^, ^102^, ^103^, ^104^, ^105^, ^106^, ^107^, ^108^, ^109^, ^110^

MRI may provide useful information regarding the early damage of spermatogenesis in testes with varicocele, possibly helping clinicians to plan appropriate treatment in men who will benefit from varicocele repair.^93^, ^94^, ^96^, ^101^, ^103^, ^104^, ^107^ A decrease in ADC has been reported in both testes with varicocele and testes contralateral to varicocele. A negative correlation between ADC and spermatic vein diameter and a positive correlation between ADC and semen analysis results has been noted in men with varicocele.^93^, ^96^, ^101^, ^103^ Moreover, FA represents another reliable parameter for the detection of testes with clinical varicocele, with an optimal cut‐off of 0.08 for the diagnosis of varicocele.^104^ Disturbances in the biochemical milieu of infertile testes with clinical varicocele have been observed. Proton MRS showed decrease in normalized concentrations of total choline, myo‐inositol, Glx complex, and lipids in testes with clinical varicocele, when compared to normal testes.^107^

Promising results have been reported regarding the potential role of functional MRI techniques, including DWI and MTI in the evaluation of male infertility. An increase in testicular ADC and a decrease in MTR have been reported in states of defective spermatogenesis.^99^, ^100^ Increase in both ADC and FA was found in testes of men with non‐obstructive azoospermia (NOA) and ADC has been shown to be a useful diagnostic parameter in identifying the subpopulation of NOA men with foci of advanced spermatogenesis up to the haploid gamete stage.^98^ More importantly, in a retrospective study of 49 NOA men, ADC and MTR proved valuable parameters to predict the probability of finding viable spermatozoa, prior to microdissection testicular sperm extraction (mTESE).^102^ Specifically, NOA testes had higher ADC and MTR when compared to normal population. A decrease in both parameters was found in NOA testes with foci of advanced spermatogenesis. On the contrary, higher ADC and MTR were observed in NOA testes with negative sperm retrieval when compared to those with positive results.^102^

Biochemical alterations in NOA testes might also be used as non‐invasive prognostic parameters for successful sperm retrieval.^92^, ^97^, ^105^, ^108^ A decrease in concentrations of total choline, total creatine, myo‐inositol, glutamate, and lipids is observed in NOA testes, when compared to normal population. Total choline represents a reliable predictor for the detection of NOA, with a cut‐off of 0.616 mmol/kg to differentiate between NOA and normal testes.^97^, ^105^, ^108^

**FIGURE 7:**
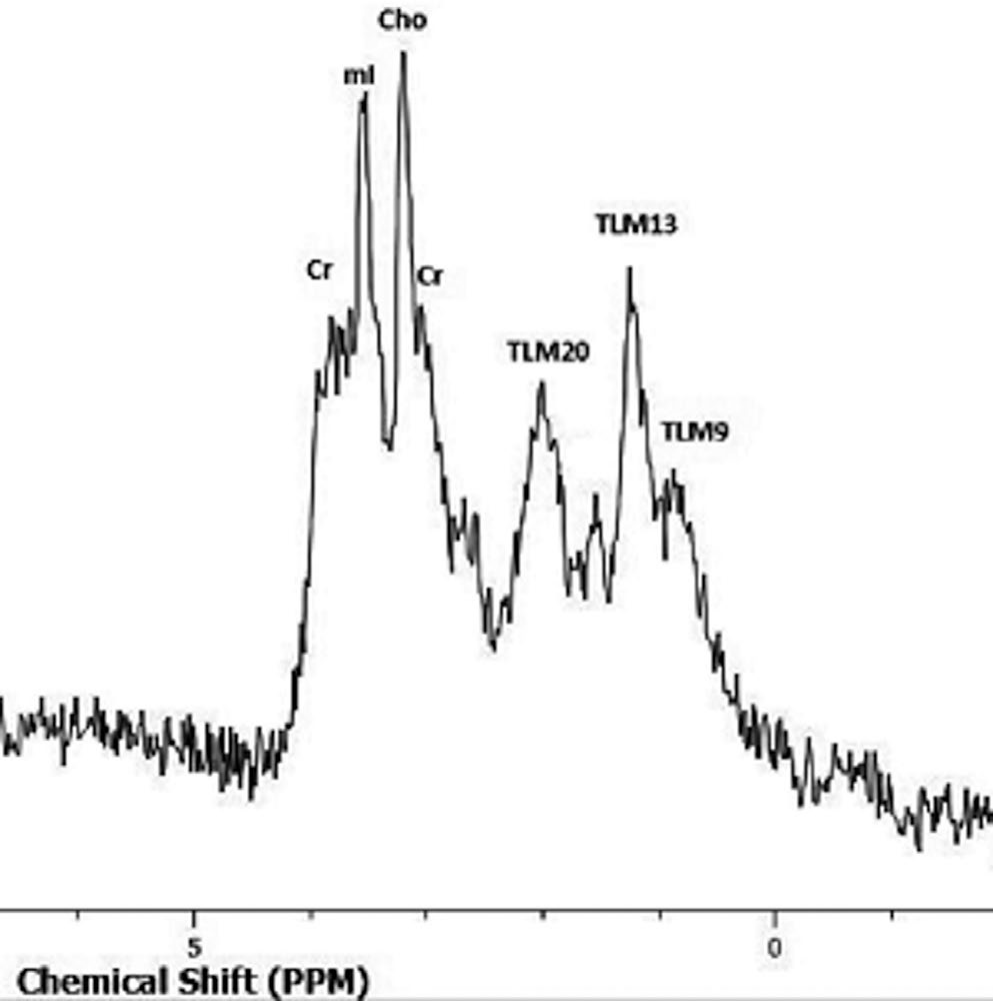
Non‐obstructive azoospermia. Proton MR spectrum of the right testis depicts decrease in levels of choline, myo‐inositol, and lipids (Cr, creatine; Cho, choline; Glx: glutamate and glutamine; mI, myo‐inositol; TLM 2.0 ppm, total lipids and macromolecules resonating at 2.0 ppm; TLM 1.3, total lipids and macromolecules resonating at 1.3 ppm; and TLM 0.9 ppm: total lipids and macromolecules resonating at 0.9 ppm). Microdissection TESE was negative for the presence of viable spermatozoa

Testicular lipids represent the most useful discriminating metabolite in the characterization of the histologic subtype of NOA. Increase in lipid peaks is seen in NOA testes with presence of foci of advanced spermatogenesis up to the haploid gamete stage.^105^ NOA testes with positive sperm retrieval often have higher choline, creatine, and myo‐inositol levels, when compared to NOA testes with negative results post‐mTESE (Figure 7). Choline proved the most sensitive metabolite in predicting the probability of finding spermatozoa, before mTESE. An increase in glutamate also has been observed in NOA testes with failed sperm retrieval.^97^, ^105^, ^108^

Furthermore, recently testicular fat deposition was measured using the fat fraction map of mDIXON Quant sequence in middle‐aged overweight patients, including infertile and normal population.^109^ The technique proved a reliable tool for the measurement of testicular fat deposition, enabling an accurate diagnosis and monitoring of male infertility.^109^

## 4 CONCLUSIONS

Although CDUS remains the primary modality for the investigation of scrotal pathologies, mpMRI may be used as a valuable diagnostic adjunct. The technique represents a tool of high diagnostic performance, providing morphologic and functional information. The main goal in scrotal imaging is to reduce the number of unnecessary radical surgical explorations. MRI greatly helps by improving scrotal lesion characterization.

Based on recommendations published by the SPIWG and review of the recent literature, scrotal MRI should be asked for the following: (1) discrimination between intratesticular and paratesticular lesions (rarely needed), (2) characterization of paratesticular and intratesticular lesions, in case of ambiguous US findings, (3) differentiation between germ cell and sex cord‐stromal testicular neoplasms, especially in case of small, non‐palpable testicular tumors, incidentally found on US, (4) pre‐operative local staging of testicular germ cell neoplasms, in candidates for testis‐sparing surgery, (5) differentiation between seminomas and non‐seminomas, when immediate chemotherapy is needed, (6) assessment of acute scrotum and scrotal trauma (in rare cases of equivocal US findings, as a complimentary examination), and (7) detection and localization of undescended testes, following uncertain US findings. Although reported preliminary data are promising, the potential role of mpMRI in the assessment of impaired spermatogenesis in infertile men is still under investigation. New and specialized MRI techniques have recently been added in the MRI protocol of the scrotum, helping us to improve our knowledge on the nature of scrotal masses and the extremely complex process of spermatogenesis.

## CONFLICT OF INTEREST

None.

## AUTHOR'S CONTRIBUTION

AT and LM conceived and designed the study. MD and GE contributed equally in the definition of the search keys, in the critical evaluation of the articles, and in the writing of the draft. AT and LM wrote the manuscript. MA and CC revised the article. AT and LM provided the final approval of the completed article.
